# The Academic Medicine and Leadership Track for Medical Students

**DOI:** 10.1007/s40670-023-01959-w

**Published:** 2023-12-15

**Authors:** Kelli Glaser, Matthew McEchron, Clyde Jensen, David Park

**Affiliations:** 1 Rocky Vista University College of Osteopathic Medicine Colorado, 8401 S. Chambers Road, Englewood, CO 80112 USA; 2 Rocky Vista University College of Osteopathic Medicine Southern Utah, Ivins, USA; 3 Rocky Vista University Montana College of Osteopathic Medicine, Billings, USA

**Keywords:** Academic medicine, Teaching, Leadership, Undergraduate medical education, Service learning

## Abstract

Physicians are expected to be educators and leaders, but few medical schools offer dedicated coursework or training to prepare medical students to meet those expectations. Since 2018, Rocky Vista University College of Osteopathic Medicine has offered a longitudinal Academic Medicine and Leadership (AML) Track in which medical students acquire knowledge and develop skills for academic medicine and leadership that will enhance their ability to become effective educators and leaders in their clinics, hospitals, professional associations, communities, and affiliated medical schools. This paper describes the novel AML Track, its learning activities, and some of its emerging outcomes.

## Background

Rocky Vista University (RVU) is an innovative and relatively new institution for health science education careers located in the mountain west region with three osteopathic medical school campuses in Colorado, Utah, and Montana. To meet the healthcare workforce demands of underserved communities and other areas of need, our institution developed a variety of career emphasis tracks for our osteopathic medical students. These tracks are sought-after elective opportunities for students with specific interests and goals to meet the needs of various communities, including those in rural areas, urban underserved areas, and globally. As medical education institutions continue to expand in recent years, it became apparent there is an increasing need for well-trained clinician medical educators who can lead in complex and diverse healthcare and educational settings. This was the impetus for the development of the Academic Medicine and Leadership Track at RVU. While some institutions have programming related to teaching students to become future educators or leaders [1, 2], only 37% span the 4 years of undergraduate medical education [1]. Similarly, a number of studies have carefully examined the various types of leadership curricula in undergraduate medical education [2–4], but few medical schools provide leadership training as a stand-alone course or a set of courses [2]. To fill this gap, RVU developed the Academic Medicine and Leadership (AML) Track in 2018 on the Utah campus, with the first cohort graduating in 2021. RVU’s Colorado campus started the AML Track program in 2022, and the Montana campus will launch the AML Track in the spring of 2024.

The AML Track enables medical students to develop leadership and teaching skills that prepare them to become effective educators and leaders in clinics, hospitals, professional associations, communities, and affiliated medical schools. Students can tailor their hands-on activities to gain the knowledge and skills required for their personal career goals. Similar to the benefits of spaced repetition for learning in medical school [5], providing AML track activities that span all 4 years and provide repeated exposures and experiences is more likely to keep students engaged and inspired to pursue careers in academic medicine and increase their confidence in stepping into leadership roles the more they practice these skills.

Overall, RVU’s AML Track is a promising development in medical education, providing students with the necessary skills and knowledge to become leaders and educators in the healthcare industry.

## Activity

Similar to all of the tracks at RVU, the AML Track spans the duration of the student’s enrollment from the second semester of their first year through graduation. Students must apply for the limited seats available in the track. There are two formal semester-long courses each consisting of 30 contact hours that run during the spring of the first year and fall of the second year. The curriculum includes a range of topics, listed in Table 1, designed to prepare students for the challenges they may face as educators and leaders such as effective communication, teaching strategies, curriculum development, and assessment methods. Furthermore, the inclusion and integration of Diversity, Equity, and Inclusion topics reflects the increasing importance of these issues in medical education, healthcare, and leadership in order to effectively address health disparities. Learning about and providing experiences for service and advocacy to address health disparities is also an important part of the curriculum. Each topic listed in Table 1 constituted 1–2 h of class time, and learning modalities integrated active learning features to promote student engagement with the material and deliberate practice.

**Table 1:** AML curricular topics and skills

**Topics and skills**	**Learning modality**
History of Osteopathic Medicine	Video Lecture
History of Medical Education	Lecture/Panel discussion
International Medical Education	Lecture; Video reflection
Graduate Medical Education	Lecture; Discussion
Organized Medicine	Lecture; Involvement with student organizations
Professional self-regulation	Lecture
Academic Leadership Roles/Responsibilities	Lecture; Breakout activity
Medical Educator Roles/Responsibilities	Lecture
Hospital System Leadership Roles	Lecture; Discussion
Curriculum Design	Lecture; Breakout activity
Course Design	Lecture; Breakout activity
Lesson Design	Lecture; Student teaching presentation assignment
Assessment Design	Lecture; Student teaching assessment assignment
Research Design	Lecture; Student project design assignment
Scholarly Writing	Lecture; Student scholarly papers
Accreditation	Lecture; Breakout activity
Personality Types	Lecture; Self-assessment; Breakout discussions
Emotional Intelligence	Lecture
Leadership Styles	Lecture; Breakout activity
Change styles and change psychology	Student lecture
Diversity, Equity, and Inclusion	Lecture; Breakout activity
Inclusive Leadership	Lecture; Breakout activity
Collaboration	Student lecture; Scholar activities
Building Teams, Coalitions, and Communities	Student lecture; Scholar activities
Coaching and Mentoring	Student lecture; Scholar activities
Conflict Management	Student lecture; Scholar activities
Feedback skills	Lecture; Repeated active practice orally and in writing
Time management	Lecture; Reflection and planning activity
Advocacy	Lecture; Advocacy efforts
Healthcare Policy	Lecture; Breakout activity
Resolution Writing	Lecture; Active practice
Public Speaking	Lecture; Active practice in class; Scholar activities
Presentation Skills	Lecture; Active practice in class; Scholar activities
Goal Setting	Lecture; Breakout activity
Strategic Planning	Lecture; Breakout activity
Conducting Meetings	Lecture; Breakout activity
Creating a Budget	Student lecture; Scholar activities
Service Learning	Student lecture; Scholar activities

The AML curriculum at RVU is designed to promote active learning and development of essential skills and knowledge for medical education and leadership. While traditional lecture presentations from medical education leaders are part of the program, the curriculum emphasizes small group breakout sessions where students can practice problem-solving and skills such as public speaking, curriculum development, and teaching activities. Additionally, students engage in activities outside class time to foster their teaching, leadership, and advocacy skills. The program provides individualized mentoring to AML Track students to assess their interests and career trajectory in medical education and leadership.

During the clinical years, classroom time is replaced by independent, self-directed learning related to teaching, research, service, leadership, and advocacy. The program monitors these self-directed tasks as “scholar activity points,” shown in Table 2, which are required for graduation from the track and to attain honors in the required courses. Scholar points can be earned throughout the entire track, but are currently the only method for engaging students in track content and activities during the clinical years. Depending on the amount of scholar points earned, students have the opportunity to earn an honors grade in the track (90 points for each AML I and II semester course) and graduate with distinction from the track (280 total points), and at least 50 points during the clinical years to graduate from the track without distinction. This method of measuring AML student involvement began in 2022 for the class of 2025 cohort. The amount of scholar points awarded for each activity was primarily based upon the estimated time a student would spend on an activity if they chose to engage in it. The AML Track stands out for its flexibility in the types of opportunities that can be used to attain these scholar activity points.

**Table 2:** AML Track scholar activities

**Scholar activities**
Attendance (> 90%)
Student Government Association or Class Officer
Club Officer
Young Doctors Steering Committee Lead
Young Doctors Steering Committee Member
Young Doctors Presenter
AML Conf. Steering Committee Lead
AML Conf. Steering Committee Member
AML Conference Attendance
Health Policy Analysis presentation
Plan & Present Virtual Grand Rounds
Attend Grand Rounds
Journal Club article presentation
Research Poster Presentation
Peer-Reviewed Journal Publication
Intercollegiate Interprofessional Education Workshop (*outside RVU courses*)
Serve on medical association board
Attend professional medical conference
House of Delegates Participant
Serve on an RVU standing committee
Serve on a Student Club Committee
Serve as an RVU Ambassador
Serve as a Tutor or Peer Mentor
RVU Fellowship (Osteopathic, Anatomy, etc.)
Lead RVU-affiliated community event
Complete formal advocacy training
Book Report or Reflection
Record AML-related instructional video
Other (must be discussed with the course director)

One example of such an activity in the realm of service learning is a collaborative program with local school districts called the Young Doctors Program (YDP), which was developed and administered by AML students. This program offers a one-semester curriculum for fifth-grade elementary school students to learn about health and health careers. Some sessions included the use of standardized patient exam rooms and simulation center which enabled the track students to gain a better understanding of how educators incorporate these activities into curriculum. The YDP provides a unique opportunity for medical students to engage with the community and inspire interest in health careers among elementary school students. By exposing young students to the world of medicine, students can gain a better understanding of the importance of health and how medical professionals contribute to their well-being. Additionally, the program introduces osteopathic medicine as a career option, increasing awareness of this field and potentially inspiring future students to pursue this career path. Second, the YDP provides AML Track students with valuable experience in teaching and mentorship, helping them develop essential skills in communication, leadership, and education. These skills are highly valuable in medical careers that involve teaching, mentoring, and leadership positions. Third, the YDP strengthens relationships between the RVU medical school and the surrounding community, promoting collaboration and mutual understanding. This type of community engagement is essential for building trust and improving health outcomes in the community. Finally, the YDP can serve as a model for other medical schools and programs interested in developing service learning opportunities for their students. The program’s success demonstrates the value of engaging with the community and promoting interest in health careers among young students. The YDP has expanded to engage multiple schools, locations, and age groups. An offshoot, Youth in Medicine, has begun on the Colorado campus that is a year-long program for high school students from communities with a high percentage of underrepresented students in medicine.

Another yearly AML student project is the creation of a leadership conference for medical students outside of AML and RVU. The conference is fully planned and implemented by AML Track students to benefit their peers who desire to gain leadership knowledge and skills.

Each year, 15–20 students are enrolled in the AML Track on each campus. Tracks have capped enrollment due to the structure of required active learning exercises and experiential opportunities. Thus, students apply near the end of their first semester of medical school by submitting essay responses to questions designed to identify future career goals and determine if their interests align with the track.

Student feedback about the AML Track has been gathered through formal course evaluations, pre/post track self-assessment surveys, and focus group discussions at the end of each semester.

## Results

To date, all students who have enrolled in the track courses have received passing grades. Student feedback suggests that active learning, public speaking exercises, and personalization are important components of the course. Self-assessments indicated growth in a variety of knowledge and skills over the course of the AML Track.

Students in the AML Track have held numerous leadership positions within RVU and nationally in various organizations, including student board positions for state and national organizations. Many participate as teaching fellows and peer tutors. They participate in advocacy events and as delegates for national meetings of various medical organizations. Nearly all of them are actively involved in ongoing research projects. Table 3 demonstrates the percentage of AML students who participated in categories of scholar activities during the fall semester of 2022 and spring of 2023. The first cohort of 18 students who graduated from this program are entering their second year of residency training and long-term outcomes are not yet available.

**Table 3:** Scholar activity participation classes of 2025 and 2026

Activity	Percent of students *N* = 67
Student government/club officer	79%
Tutoring/mentoring	48%
Young doctors’ presentations	58%
Professional medical conference attendance	52%
Standing school committees/leadership steering committees	43%
Research poster presentations or journal publication	21%
Community board member/ambassador/leader	21%
Advocacy training/advocacy presentations	12%
Medical association board member	1%

## Discussion

The educational significance of this Track is that students can tailor their hands-on activities to allow them to gain the knowledge and skills required to succeed as future educators, leaders, and advocates in the medical profession.

The value of the AML Track can be assessed through student feedback and their achievements. Student feedback has been very positive on the surveys and the doubling of applications for the AML Track reflects its growing popularity. In Colorado, course directors found that focus groups were the most helpful form of feedback as students were more apt to provide suggestions for changes when compared with a formal course evaluation process. One of the changes made to the track as a result of this feedback was the addition of more active learning modalities. Another change being explored is mechanisms to offer additional experiences for students including credit bearing courses during the clinical years.

One of the limitations of the AML Track is the challenge of staying connected to students while they are away on clinical rotations, as there is no formal credit-bearing required courses for the AML Track during years 3 and 4. This is something being explored for the future and we believe would further emphasize AML skill development. Another challenge will be accurately gathering the achievements of track graduates through residency and beyond in order to gauge the impact of AML on our students’ career trajectories and professional development. Administration of longitudinal surveys is one approach being considered.

Overall, the AML Track at RVU provides a unique and comprehensive program that emphasizes active learning and development of essential skills for medical education and leadership. The program’s emphasis on flexible self-directed learning ensures that students have a customized and relevant experience that prepares them for future success in their careers. We believe that our model may provide a feasible example for institutions of medical education who desire to foster the development of future medical educators and leaders.

## Data Availability

Raw data analyzed during this study are not included in this published article in order to protect the anonymity of the small population of student participants who might be identifiable through the combination of their reported activities used to create Table 3.
